# Life-Stage-Specific Prioritization of Feline Diseases for Health Screening in South Korea

**DOI:** 10.3390/ani16213400

**Published:** 2026-10-28

**Authors:** Yu-Ha Kim, Yoon Jung Do, Kyung-Duk Min

**Affiliations:** 1 Laboratory of Veterinary Epidemiology, College of Veterinary Medicine, Chungbuk National University, Cheongju 28644, Republic of Korea; k46561336@gmail.com; 2 Animal Diseases & Health Division, National Institute of Animal Science, Rural Development Administration, Wanju 55365, Republic of Korea

**Keywords:** feline health screening, veterinary guidelines, priority diseases, diagnostic proportion, clinical importance

## Abstract

Feline health screening in South Korea lacks local disease priorities. We developed a model combining diagnostic proportions from 39,016 diagnosis entries with disease importance and treatment/management efficacy ratings from 39 veterinarians. Two scoring versions used different orders of multiplication and decile conversion across four life stages: kitten, young adult, mature adult, and senior. Eligibility required a stage-specific diagnostic proportion of at least half the overall proportion for that disease and at least 0.3% of diagnoses in that stage. The versions produced similar top-10 lists. Stomatitis was selected for all four life stages; stomatitis and six additional diseases—chronic kidney disease, urolithiasis, systemic hypertension, pancreatitis, diabetes mellitus, and periodontal disease—were selected for all three adult stages. Infectious diseases featured prominently in kittens, whereas chronic and metabolic diseases became prominent in older cats. Hyperthyroidism was selected only in seniors, and neoplasia remained below the top 10, reflecting its low efficacy score. Scoring prioritized severe chronic or life-threatening conditions more highly than diagnostic proportions alone. Diagnostic proportions describe recorded diagnoses rather than individual-cat population prevalence. These candidate lists require validation and evaluation of screening tests, clinical benefits, cost, accessibility, and patient stress before guidelines can be established.

## Simple Summary

Regular health checks can help identify health problems in cats, but deciding which diseases deserve attention at different ages can be difficult. We developed a method to identify potential priorities for feline health screening in South Korea. We combined disease information from veterinary hospital records with assessments by 39 veterinarians of disease severity and the expected benefits of treatment and management. Screening priorities were calculated separately for kittens, young adults, mature adults, and seniors. Two scoring approaches produced broadly similar selections. Infectious diseases featured prominently in kittens, while chronic and metabolic diseases became more prominent in adults and seniors. Stomatitis was selected at every life stage, and hyperthyroidism was selected only in seniors. These findings provide a starting point for developing screening recommendations that reflect local veterinary practice. The selected diseases require further evaluation of suitable tests, costs, accessibility, and stress to the cat before practical screening recommendations can be established.

## 1. Introduction

The companion animal population in South Korea is expanding rapidly. Specifically, the companion cat population increased by 22.6%, from 1.77 million in 2020 to 2.17 million at the end of 2024 [1]. As cats are increasingly recognized as integral members of families, public demand for accurate and validated feline health screening has increased. Despite this growing need, veterinary practice in South Korea still relies on unsystematic screening protocols that may not meet evolving clinical needs.

In countries with advanced veterinary healthcare systems, such as the UK, the US, and Sweden, health screening guidelines and disease prevalence data are increasingly incorporated into clinical practice [2,3,4,5,6,7,8,9]. However, these guidelines have generally been developed for individual diseases or clinical domains, and the criteria used to prioritize diseases for screening remain largely unclear. Existing guidance addresses specific clinical domains, including the management of hyperthyroidism [10], dental care [11], and feline vaccination [12], while a separate review describes a systematic approach to oral examination [13]. Although feline life-stage guidelines lack the epidemiological evidence needed to justify the conditions selected for screening and the recommended screening intervals [14], frameworks that jointly consider diagnostic proportions and clinical importance have rarely been applied to feline health screening.

South Korea currently has no consensus-based feline health screening guidelines that reflect its clinical and social contexts. The literature on feline health in South Korea has mostly been limited to case-based evaluations of specific diagnostic methods, such as the antemortem diagnosis of feline infectious peritonitis (FIP) [15], while fragmented and insufficiently standardized veterinary medical records limit the generation of representative epidemiological evidence [16] needed for data-driven prioritization of diseases. Moreover, guidelines developed in other countries may not be directly applicable to South Korea because breed composition, living environments, and patterns of veterinary care differ across countries.

Therefore, this study aimed to propose a systematic disease-prioritization model tailored to veterinary practice in South Korea. We stratified feline patients into four life stages (kitten, young adult, mature adult, and senior) and identified the 10 highest-scoring eligible candidate diseases for each stage. As no consensus has been established regarding how diagnostic proportion should be combined with clinical importance and treatment/management efficacy, we compared two score-aggregation procedures as a first step toward systematic disease prioritization. These candidate lists are intended to inform the development and evaluation of feline health screening recommendations in South Korea.

## 2. Materials and Methods

### 2.1. Study Design

Priority diseases were identified by calculating a score for each candidate disease within each life stage and selecting the 10 highest-scoring eligible diseases. The score combined diagnostic proportion derived from electronic medical records (EMR) with two expert-derived criteria: disease importance and treatment/management efficacy. Scores were calculated for all candidate categories before the life-stage eligibility criteria were applied.

### 2.2. Data Source, Life Stages and Diagnostic Proportions

The feline EMR data were obtained from the Korean veterinary EMR resource described by Park et al. [17]. The archived extract used here contained records dated January 2020 through December 2022 from 27 veterinary hospitals. Although the source publication reports a collection period of 2020–2023, no 2023 records were present in this extract; the dates and counts reported here refer to the verified extract. Data were provided in de-identified form under an agreement requiring that participating hospitals not be identifiable, and hospitals were represented by codes. The vendor was Woorien Co., Ltd. (Hwaseong, Republic of Korea). The available extract and documentation did not identify the exact software product or provide a verified regional and care-level breakdown of the 27 hospitals.

In the source study, free-text clinical records were translated into English and diagnostic terms were extracted using GPT-4o. Terms were standardized to Small Animal Problem and Diagnosis Terms (SA-PDT), a veterinary subset of SNOMED CT, using language-model matching and veterinary review [17]. The present analysis used the archived, reviewed diagnostic-term mapping and grouped related diagnoses into the prespecified candidate disease categories.

The archived feline extract contained 207,822 diagnostic-term rows. Applying the archived mapping retained 44,441 mapped rows and excluded 163,381 without a matching term. Of the mapped rows, 868 had no final category, 968 were assigned to symptoms, and 2695 to findings. Excluding these rows left 39,910 diagnosis counts. We then removed 894 duplicate rows with identical hospital–visit–patient identifiers (site_visit_pet_id) and original diagnostic-term text (post_processing), leaving 39,016 diagnosis counts for analysis. Different terms assigned to the same category within a visit and repeated diagnoses on separate visits were retained; each disease was not reduced to one occurrence per cat. These term-level counts and exclusions are distinct from numbers of visits or cats.

Age was expressed in completed years. The life stages were kitten, 0–1 years (younger than 2 years); young adult, 2–7 years (2 to younger than 8 years); mature adult, 8–11 years (8 to younger than 12 years); and senior, 12 years or older. The source study’s category labels of 1–2, 3–8, 9–12, and 13 or older corresponded to these groups, with labels one unit greater than completed age.

The retained entries arose from 26,603 visits and 11,904 unique hospital–cat identifier combinations. In kittens, young adults, mature adults, and seniors, respectively, diagnosis counts were 11,965, 16,022, 7098, and 3931; hospital-specific cat counts were 4984, 4998, 1611, and 698; and visit counts were 8933, 10,905, 4436, and 2329. Cats could contribute to more than one life stage as they aged, so stage-specific cat counts cannot be summed to obtain the overall number. No shared animal-level identifier was available across hospitals. We therefore could not reliably identify and exclude records for cats attending multiple hospitals, and the 11,904 hospital–cat combinations do not necessarily represent 11,904 distinct cats across hospitals.

For each disease category and life stage, the diagnostic proportion was calculated as n/N, where n is the number of retained diagnosis entries assigned to that category, and N is the total number of retained diagnosis entries in that stage. Denominators included all retained diagnostic categories, not only the 32 prioritization candidates. Counts for related diagnoses were combined using the existing candidate-category definitions. The diagnostic proportion describes the composition of recorded diagnoses, not individual-cat prevalence or incidence.

Stomatitis was operationally defined as recorded inflammatory or ulcerative diagnoses involving the oral mucosa, gingivae, lips, or tongue. The category comprised seven terms: stomatitis, gingivostomatitis, lymphocytic plasmacytic gingivostomatitis, cheilitis, lip ulcer, ulcer on tongue, and ulceration of gingivae. Grouping was based on anatomical and lesion similarity, without requiring a common cause, chronicity, or histopathological confirmation; it was therefore broader than feline chronic gingivostomatitis. These terms contributed 1226 entries: 285, 611, 207, and 123 in the four successive life stages. Dental calculus and plaque, tooth resorption, and periodontal disease labels were assigned to a separate dental/periodontal category comprising 2588 entries. The categories separated diagnosis labels, not mutually exclusive groups of cats; a cat could have both types of diagnosis.

### 2.3. Expert Survey and Category-Level Scores

An expert survey was conducted from August 1 to September 15, 2025, targeting clinical professors at veterinary colleges and directors of veterinary clinics. Thirty-nine practicing clinical veterinarians participated. Respondents were asked to select diseases they considered clinically important and suitable for preventive health screening and then rate the selected diseases. The present analysis used their “High,” “Medium,” or “Low” ratings of disease importance and treatment/management efficacy. The number of respondents varied across diseases.

Disease importance was defined based on the financial burden imposed on the caregiver at diagnosis and the impact of the condition on the patient’s quality of life and lifespan. “High” denoted life-threatening conditions or those causing severe functional impairment that, without appropriate intervention, would lead to death or a marked deterioration in quality of life; “Medium” denoted conditions that are not immediately life-threatening but cause considerable discomfort and result in long-term adverse effects if treatment is delayed; and “Low” denoted mild and largely self-limiting conditions.

Treatment/management efficacy was defined as the potential for prognostic improvement with early detection and the availability of effective treatments in general veterinary practice. “High” denoted conditions for which early detection offers a high probability of complete cure or substantially slows progression and for which standard treatments are readily available; “Medium” denoted conditions for which intervention is effective in controlling symptoms and slowing progression but does not achieve cure; and “Low” denoted conditions with limited prognostic benefit from early detection, no accessible treatment, or low efficacy relative to cost and adverse effects.

Responses were assigned 15, 10, and 5 points for “High,” “Medium,” and “Low,” respectively. Category-level scores were calculated from rounded item-specific response percentages. For each item and criterion, the percentage-weighted mean was (15 × High percentage + 10 × Medium percentage + 5 × Low percentage)/100; multiplying this mean by 10/15 expressed it on a 10-point scale. Scores for the 46 questionnaire items used in this analysis were consolidated into 32 disease categories. For categories represented by multiple items, the rescaled item scores were averaged with equal weight across items. Category-level scores were rounded to two decimal places before decile conversion.

Individual responses were used to calculate item-level rating distributions and descriptive statistics; category-level model scores were calculated from the rounded response percentages described above. Respondents were anonymized as V01–V39. Across the 46 source items, there were 1007 valid ratings for each of the two criteria, with item-specific valid counts ranging from 4 to 38. Supplementary Table S3 reports individual scores, High/Medium/Low counts, valid response counts, means, and sample standard deviations on the original 15/10/5 scale; standard deviations used n−1 in the denominator. All observed ratings were included. Unrecorded ratings were treated as missing, not as zero, and were not imputed. Item-level descriptive statistics are reported separately from the category-level model scores in Supplementary Table S2.

### 2.4. Priority Score Calculation and Eligibility

Two scoring versions combined diagnostic proportion with the category-level disease importance and treatment/management efficacy scores (Table 1). Decile conversion placed the components on a common rank scale, and multiplication allowed each component to influence the combined score, with a low component reducing the product. The formulas were not derived from statistical hypothesis tests, and no component weights were estimated from outcome data.

Decile conversion used all 32 candidate categories before the life-stage eligibility filters were applied. Values were ranked in ascending order, ties received their average rank, and the decile score was the ceiling of (10 × average rank/32), giving scores from 1 to 10. Diagnostic proportion deciles were calculated separately within each life stage; expert-derived deciles were common to all stages.

In Version 1, the importance, efficacy, and diagnostic proportion deciles were multiplied. In Version 2, the rounded category-level importance and efficacy scores were multiplied first; this product was converted to a decile score and multiplied by the diagnostic proportion decile. The versions were compared to examine how the order of aggregation and decile conversion affected the selected diseases.

The age-specific-to-overall diagnostic proportion ratio was calculated by dividing the stage-specific diagnostic proportion for a disease category by its diagnostic proportion across all life stages. Each proportion used the corresponding disease-category count divided by the total diagnosis count. This compares the composition of recorded diagnoses; it is not a relative risk or an incidence-rate ratio. After scores were calculated for all 32 candidate categories, a disease was eligible only if this ratio was at least 0.5 and its stage-specific diagnostic proportion was at least 0.003 (0.3%). Both thresholds were applied to unrounded values. Ineligible diseases were removed from the stage-specific list without recalculating scores.

Eligible diseases were ordered by descending priority score. Equal final scores were ordered by the Korean disease-category labels in ascending order under the Korean locale, after which sequential positions were assigned. The first 10 eligible diseases were selected for each life stage and scoring version. This deterministic rule defined membership and display order without implying a clinical distinction between diseases with equal scores.

### 2.5. Confidence Intervals and Software

For each candidate category, pointwise 95% confidence intervals for diagnostic proportions were estimated by a nonparametric hospital-cluster bootstrap. In each of 10,000 replicates, 27 hospitals were sampled with replacement, and all retained diagnosis entries from each sampled hospital were carried together. The disease count and diagnostic denominator were recomputed jointly, and the 2.5th and 97.5th percentiles of the resulting proportions formed the interval. This preserved dependence among diagnoses, visits, and cats within a hospital while treating hospitals as independent sampling units. Every replicate had a positive denominator in every stage of life. When a disease had no observed diagnoses in any hospital for a stage, an informative nonparametric bootstrap interval could not be estimated and was marked NE. The intervals were descriptive and did not alter eligibility or scoring.

All analyses were performed using R version 4.5.1 (R Core Team, Vienna, Austria).

## 3. Results

The analytical dataset contained 39,016 diagnosis entries from 26,603 visits and 11,904 unique hospital–cat identifier combinations across 27 hospitals. The stage-specific diagnosis counts were 11,965 in kittens, 16,022 in young adults, 7098 in mature adults, and 3931 in seniors. Stomatitis accounted for 2.38%, 3.81%, 2.92%, and 3.13% of the respective diagnostic denominators, while hyperthyroidism accounted for 0.01%, 0.02%, 0.37%, and 2.16%. These diagnostic proportions describe recorded diagnoses, not the percentages of individual cats affected.

The survey included 39 veterinarians working at primary and secondary veterinary clinics and university veterinary teaching hospitals, 77% of whom had more than 10 years of clinical experience. Their specialties were predominantly internal medicine and general practice, followed by dermatology and surgery. Their caseloads consisted mainly of dogs and cats, with exotic species accounting for a negligible proportion. The 46 source questionnaire items contributing to the priority model yielded 1007 valid ratings for disease importance and 1007 for treatment/management efficacy, with 4–38 valid ratings per item. Supplementary Table S3 presents the anonymized individual ratings from all 39 respondents, item-specific High/Medium/Low counts, valid response counts, means, and sample standard deviations for both criteria. The category-level scores used for prioritization are reported separately in Supplementary Table S2.

The 10 selected candidate diseases for each life stage under Versions 1 and 2 are listed in Table 2, with their scores in Supplementary Table S1. Supplementary Table S2 reports diagnosis counts, diagnostic proportions with hospital-cluster bootstrap 95% confidence intervals, eligibility status, and both priority scores for all 32 candidate categories. The two versions showed high consistency. The lists were identical in composition for the kitten and mature adult stages and shared 9 of the 10 diseases for the young adult and senior stages, although their order differed. In the young adult and senior stages, hypertrophic cardiomyopathy was selected under Version 2 in place of hydronephrosis and hepatic lipidosis, respectively, which were selected under Version 1. Stomatitis was the only disease selected for all four life stages in both versions. Stomatitis and six additional diseases—chronic kidney disease, urolithiasis, systemic hypertension, pancreatitis, diabetes mellitus, and periodontal disease—were selected for all three adult stages in both versions. Hypertrophic cardiomyopathy and pancreatitis generally occupied higher positions in Version 2, whereas diabetes mellitus and stomatitis occupied higher positions in Version 1. The composition of the candidate lists shifted with age from infectious and early-onset conditions to chronic and metabolic diseases.

In the young adult stage, chronic kidney disease and urolithiasis ranked first and second, respectively, in both versions, and none of the infectious diseases selected in the kitten stage appeared on the list. Pancreatitis (fourth and third in Versions 1 and 2, respectively), systemic hypertension (sixth and fourth), hepatic lipidosis (fifth and seventh), diabetes mellitus (seventh and 10th), and periodontal disease (10th and ninth) entered the list at this stage, whereas feline idiopathic cystitis was retained (eighth and fifth). Hydronephrosis (ninth in Version 1) and hypertrophic cardiomyopathy (sixth in Version 2) were the only diseases that differed between the two versions.

In the mature adult stage, the two versions selected an identical set of 10 diseases. Diabetes mellitus, chronic kidney disease, and pancreatitis occupied the top three positions in Version 1, whereas chronic kidney disease, pancreatitis, and diabetes mellitus occupied the top three positions in Version 2. Systemic hypertension ranked fourth in both versions. Heart failure first entered the lists at this stage, ranking seventh in Version 1 and sixth in Version 2. Hypertrophic cardiomyopathy first entered the list at this stage under Version 1, ranking 10th; under Version 2, it was already selected in young adults and ranked seventh in mature adults. Feline idiopathic cystitis was not selected in either version.

In the senior stage, chronic kidney disease, diabetes mellitus, pancreatitis, and systemic hypertension remained among the top five diseases in both versions. Hyperthyroidism, which was not selected at any earlier life stage, ranked fifth in Version 1 and third in Version 2. Heart failure was retained (eighth and 10th), whereas hepatic lipidosis (ninth in Version 1) and hypertrophic cardiomyopathy (sixth in Version 2) were the only diseases that differed between the two versions. Neoplasia was not selected at any life stage and ranked 11th to 15th in the mature adult and senior stages in both versions.

## 4. Discussion

This study identified candidate diseases for feline health screening in South Korea by combining EMR-derived diagnostic proportions with expert-rated disease importance and treatment/management efficacy. Stage-specific eligibility criteria were applied to the scored candidates before selecting the lists. The two scoring versions produced broadly similar selections. Stomatitis was selected for all four life stages, while stomatitis and six additional diseases—chronic kidney disease, urolithiasis, systemic hypertension, pancreatitis, diabetes mellitus, and periodontal disease—were selected for all three adult stages. The kitten lists consisted of stomatitis and five infectious diseases, along with feline idiopathic cystitis, gastroenteritis, ocular disease, and dermatitis. The composition of the lists therefore shifted with age from infectious and early-onset conditions to chronic and metabolic diseases.

The age-related composition of the candidate lists is consistent with several epidemiological observations from other countries. FIP occurs predominantly in cats under 2 years of age [18]. The prevalence of chronic kidney disease in UK primary care increased from 1.2% in cats under 9 years of age to 3.6% in cats aged 9 years or older [19]. Age is a risk factor for diabetes mellitus [20], and cardiomyopathy has been identified in approximately 15% of apparently healthy cats [21]. These comparisons provide clinical context, although the populations and measures differ from the diagnostic proportions used here.

Hyperthyroidism is concentrated in cats aged 10 years or older [22]. Historical US data from nine veterinary teaching hospitals documented an increase in hospital prevalence from 0.61 per 1000 visits in 1978–1982 to 29.64 per 1000 visits in 1993–1997 (2.96%; 95% CI, 2.82–3.11%); an increase remained after age adjustment [23]. In Hong Kong, 3.93% of 305 cats aged ≥10 years sampled in 2006–2007 were hyperthyroid [24]. In Warsaw, hyperthyroidism was diagnosed in 20.14% of 417 cats aged ≥7 years examined in 2007–2011 [25]. A study in the greater Dublin area conducted in 2011–2012 identified hyperthyroidism in 21.1% of 507 cats aged ≥10 years undergoing blood sampling for routine clinical investigations [26]. A UK study published in 2026, based on health screening conducted in 2018–2023, identified hyperthyroidism in 89 of 464 evaluable cats (19.2%; 95% CI, 15.9–23.0%) within a cohort of 549 cats aged ≥8 years [27]. These studies support the relevance of hyperthyroidism to screening in older cats. However, differences in age thresholds, clinical selection, diagnostic procedures, and observation periods prevent interpreting their estimates as a single international temporal trend. Its selection only in the senior stage in the present study is consistent with its clinical importance in older cats but does not establish an increase in Korean population prevalence or show that such an increase is independent of geographic location.

The composition of each list also reflects the contribution of the three scoring components. FIP ranked highly in kittens because its high diagnostic proportion and top-decile importance outweighed its low efficacy score. Stomatitis and periodontal disease were selected for every adult stage because their diagnostic proportion and efficacy scores contributed to their overall priority scores. The broad stomatitis grouping was not restricted to feline chronic gingivostomatitis. The efficacy score reflects clinicians’ ratings of the general survey label and does not establish treatment success rates for individual oral disorders, particularly refractory feline chronic gingivostomatitis. Chronic kidney disease and diabetes mellitus combined high importance and efficacy scores with greater diagnostic proportions in older cats, while hyperthyroidism was selected when its diagnostic proportion increased in the senior stage.

The higher position of hypertrophic cardiomyopathy in Version 2 reflects the difference between the scoring approaches. Version 2 multiplies the expert-derived importance and efficacy scores before converting the product to deciles, whereas Version 1 converts each component to deciles separately. In this dataset, the very high importance score for hypertrophic cardiomyopathy offset its lower efficacy score more strongly under Version 2. This difference illustrates how the order of aggregation and decile conversion can affect the model output.

Neoplasia was not selected for any life stage, although it is among the leading causes of death in older cats [28] and accounted for 1.91% of senior-stage diagnoses in the present study, approximately three times its diagnostic proportion across all life stages. Its relatively low treatment/management efficacy score contributed to its position below the top 10. The model combined scores from eight tumor-related questionnaire items into one neoplasia category, giving each item equal weight. This aggregation combined tumors with different prognoses and treatment options, obscuring distinctions that may matter for screening. Several common feline malignancies, such as oral squamous cell carcinoma, are often managed palliatively rather than with curative treatment [29]. Perceived access to advanced oncological treatment in general veterinary practice in South Korea may also have influenced the efficacy ratings. Because early detection improves outcomes for some cancers, notably mammary carcinoma [30], future prioritization by tumor type could identify screening candidates that are not apparent within the aggregated category.

The eligibility criteria provided a pragmatic filter for diseases with a low diagnostic proportion within a life stage, either in absolute terms or relative to their diagnostic proportion across all life stages. They did not establish whether an individual diagnosis was correct. Their most conspicuous effects on the candidate lists occurred in kittens. Without the criteria, systemic hypertension would have ranked second under Version 1 and fifth under Version 2 in kittens, and chronic kidney disease, urolithiasis, and pancreatitis would also have entered the lists. Each of these conditions had a kitten diagnostic proportion less than half its diagnostic proportion across all life stages; the ratio for systemic hypertension was approximately 0.35. Hypertension is predominantly a disease of older cats, with routine screening recommended from 7 to 9 years of age [31,32], and clinical blood pressure readings can be elevated by the white-coat effect [33]. Situational elevations could contribute to some recorded diagnoses, but the available data could not distinguish these from sustained hypertension. Systemic hypertension remained in the young adult lists, with a diagnostic proportion approximately 0.8 times its diagnostic proportion across all life stages. The upper age range of this stage overlaps with the age at which blood pressure screening is recommended.

Large primary care studies in the UK and US have consistently identified dental disease, obesity, flea infestation, and dermatological or gastrointestinal disorders among the most commonly recorded feline conditions [8,34,35]. Rankings based on occurrence alone may therefore differ from lists that also consider clinical consequences and treatment options. By weighting disease importance and treatment/management efficacy alongside diagnostic proportion, the present model assigned high priority to severe chronic or life-threatening diseases, including chronic kidney disease, systemic hypertension, and FIP. The broader dermatitis and ocular disease categories were selected only in kittens. These differences should not be interpreted as direct comparisons of disease prevalence because the underlying populations, diagnostic categories, and denominators differ. Living environment, pathogen exposure, and vaccination coverage may also affect disease patterns. Outdoor access has been associated with nearly threefold higher odds of parasitic infection in cats [36], and vaccination practices vary between regions [37]. Priority lists therefore require evaluation in the clinical setting in which they will be used.

Several limitations should be considered when interpreting these candidate lists. The scores incorporated diagnostic proportion, disease importance, and treatment/management efficacy, but did not separately incorporate the potential for early diagnosis or diagnostic practicality, including test accessibility, cost, and patient stress. A high score therefore does not establish a feasible or beneficial screening target. FIP illustrates this distinction because no single noninvasive test can confirm the diagnosis in an asymptomatic cat [38,39]. Efficacy scores also reflected Korean clinicians’ perceptions at the time of the survey. Antiviral studies have reported success rates of approximately 80–90% for GS-441524-based treatment, depending on the regimen and study population [40]; evolving evidence and access to treatment may change clinicians’ assessments. The diagnostic proportions describe the composition of recorded clinical activity rather than individual-cat prevalence or incidence. Although exact duplicate diagnostic terms within a visit were removed, repeated diagnoses across visits and different terms assigned to one category within a visit were retained. Cats attending frequently and diseases requiring repeated follow-up may therefore contribute disproportionately to the diagnosis counts and priority scores. Patient identifiers were hospital-specific, and no shared animal-level identifier was available for cross-hospital linkage. The same cat could consequently be represented under more than one hospital-specific identifier. Referral patterns, coding practices, and the grouping of diagnoses may further affect the results. The data-provision agreement required that participating hospitals not be identifiable, and hospitals were represented only by codes. The exact EMR product and a verified regional and care-level profile of the 27 hospitals were unavailable in the supplied data and documentation. Differences by EMR product, region, or hospital type could therefore not be assessed, limiting evaluation of how well the participating hospitals represent veterinary practice across South Korea. The hospital-cluster bootstrap preserves within-hospital dependence, but its intervals are approximate and conditional on the observed hospitals. It cannot correct selective hospital participation, unknown attendance by the same cat at multiple hospitals, or uncertainty in diagnosis coding and category assignment. These intervals describe uncertainty in the recorded diagnostic proportions, not in individual-cat population prevalence or the final priority rankings. An NE interval for a zero observed count does not imply that the disease is absent from the population. The 39 veterinarians were a convenience sample, and the number of valid ratings varied across diseases. Selection based on perceived clinical importance and suitability for preventive health screening may influence the expert-derived scores. For categories combining several questionnaire items, equal weighting of item means does not ensure equal contributions from individual veterinarians. The anonymized individual ratings and item-level sample standard deviations make the observed variation transparent, but they do not quantify uncertainty in the final priority rankings. The eligibility thresholds were chosen empirically and were not validated against clinical outcomes. A threshold of 1.0 for the age-specific-to-overall diagnostic proportion ratio would exclude common conditions such as periodontal disease from the senior stage, so the less stringent threshold of 0.5 was retained. Decile conversion also discards the magnitude of differences between diseases, and the deterministic tie rule does not distinguish their clinical value. The numerical order describes the scoring model output; the selected diseases are candidates for further assessment and should not be interpreted as a clinically validated hierarchy or a recommended sequence of screening tests.

## 5. Conclusions

Combining diagnostic proportions from veterinary records with expert assessments of disease importance and treatment/management efficacy identified candidate diseases for feline health screening in South Korea. The two scoring approaches produced broadly similar selections, with infectious diseases prominent in kittens and chronic and metabolic diseases increasingly prominent in older cats. These findings support considering life stage when developing screening recommendations. The lists require further validation because diagnostic coding, disease grouping, and selective expert participation may influence the results. Future work should include more hospitals, seek broader expert consensus, and evaluate screening test performance, clinical benefit, accessibility, cost, and patient stress before translating these candidate lists into practical screening guidelines.

## Figures and Tables

**Table 1:** Priority Score Calculation Formulas.

Version	Formula
1	Decile [disease importance] × Decile [treatment/management efficacy] × Decile [diagnostic proportion]
2	Decile [(disease importance × treatment/management efficacy)] × Decile [diagnostic proportion]

Note: Decile [·] denotes rank-based conversion to scores from 1 to 10 among all 32 candidate categories. Diagnostic proportion deciles were calculated separately within each life stage. Expert-derived category scores, life-stage eligibility criteria, and tie handling are defined in Materials and Methods.

**Table 2:** Candidate Priority Feline Diseases by Life Stage and Scoring Version.

Version	Life Stage			
	Kitten	Young Adult	Mature Adult	Senior
1	Stomatitis	Chronic kidney disease	Diabetes mellitus	Diabetes mellitus
	Feline panleukopenia virus infection	Urolithiasis	Chronic kidney disease	Chronic kidney disease
	Feline idiopathic cystitis	Stomatitis	Pancreatitis	Systemic hypertension
	Feline infectious peritonitis	Pancreatitis	Systemic hypertension	Pancreatitis
	Gastroenteritis	Hepatic lipidosis	Stomatitis	Hyperthyroidism
	Ocular disease	Systemic hypertension	Urolithiasis	Stomatitis
	Feline calicivirus infection	Diabetes mellitus	Heart failure	Urolithiasis
	Feline herpesvirus infection	Feline idiopathic cystitis	Hepatic lipidosis	Heart failure
	Feline leukemia virus infection	Hydronephrosis	Periodontal disease	Hepatic lipidosis
	Dermatitis	Periodontal disease	Hypertrophic cardiomyopathy	Periodontal disease
2	Stomatitis	Chronic kidney disease	Chronic kidney disease	Chronic kidney disease
	Feline infectious peritonitis	Urolithiasis	Pancreatitis	Pancreatitis
	Feline idiopathic cystitis	Pancreatitis	Diabetes mellitus	Hyperthyroidism
	Feline panleukopenia virus infection	Systemic hypertension	Systemic hypertension	Diabetes mellitus
	Gastroenteritis	Feline idiopathic cystitis	Urolithiasis	Systemic hypertension
	Ocular disease	Hypertrophic cardiomyopathy	Heart failure	Hypertrophic cardiomyopathy
	Feline leukemia virus infection	Hepatic lipidosis	Hypertrophic cardiomyopathy	Periodontal disease
	Feline calicivirus infection	Stomatitis	Hepatic lipidosis	Stomatitis
	Dermatitis	Periodontal disease	Stomatitis	Urolithiasis
	Feline herpesvirus infection	Diabetes mellitus	Periodontal disease	Heart failure

Note: The 10 selected candidate diseases are presented in descending order of the model-derived priority score within each life stage and scoring version. The completed-age groups are kitten, 0–1 years; young adult, 2–7 years; mature adult, 8–11 years; and senior, ≥12 years. The scoring versions are defined in Table 1. Equal final scores were ordered by the Korean disease-category labels in ascending order, as specified in Materials and Methods. This ordering describes the model output and does not establish a clinically validated hierarchy or a recommended sequence of screening tests. In the kitten stage, the two versions selected the same 10 diseases, with stomatitis ranking first in both. Five infectious diseases, namely, feline panleukopenia virus (FPV) infection, FIP, feline calicivirus infection, feline herpesvirus infection, and feline leukemia virus infection, were selected only at this stage. FPV infection and FIP ranked second and fourth under Version 1 and fourth and second under Version 2, respectively. Feline idiopathic cystitis ranked third, whereas gastroenteritis, ocular disease, and dermatitis were included in both versions.

## Data Availability

The datasets used and/or analysed during the current study are available from the corresponding authors upon reasonable request. Access to EMR-derived data is subject to the data-provision conditions that protect participating hospitals from identification.

## Supplementary Materials

The following supporting information can be downloaded at: https://www.mdpi.com/article/10.3390/ani16213400/s1. Table S1: Top 10 Priority Feline Diseases and Calculated Scores by Life Stage; Table S2: Complete Candidate Diagnostic Proportions and Priority Scores; Table S3: Individual Expert Ratings and Descriptive Statistics.
