# Supplementary material for: Life-Stage-Specific Prioritization of Feline Diseases for Health Screening in South Korea
Source: Animals (Basel). 2026 Oct 28;16(21):3400. doi: 10.3390/ani16213400 (PMC13761872; doi:10.3390/ani16213400)
Supplement: Supplementary file 1 [file animals-16-03400-s001.zip › animals-4607721-supplementary.pdf]

animals

MDPI

Supplementary Table S1. Top 10 Priority Feline Diseases and Calculated Scores by Life Stage.

Version	Score by Life stage			
1[1]	**Kitten**	**Score**	**Young adult**	**Score**
	Stomatitis	450	Chronic kidney disease	600
	Feline panleukopenia virus infection	350	Urolithiasis	512
	Feline idiopathic cystitis	336	Stomatitis	450
	Feline infectious peritonitis	320	Pancreatitis	448
	Gastroenteritis	216	Hepatic lipidosis	441
	Ocular disease	200	Systemic hypertension	432
	Feline calicivirus infection	120	Diabetes mellitus	405
	Feline herpesvirus infection	108	Feline idiopathic cystitis	378
	Feline leukemia virus infection	98	Hydronephrosis	300
	Dermatitis	90	Periodontal disease	300
	**Mature adult**	**Score**	**Senior**	**Score**
	Diabetes mellitus	648	Diabetes mellitus	648
	Chronic kidney disease	600	Chronic kidney disease	600
	Pancreatitis	560	Systemic hypertension	540
	Systemic hypertension	486	Pancreatitis	504
	Stomatitis	450	Hyperthyroidism	480
	Urolithiasis	448	Stomatitis	405
	Heart failure	441	Urolithiasis	320
	Hepatic lipidosis	441	Heart failure	315

[1] Decile [disease importance] × Decile [treatment/management efficacy] × Decile [diagnostic proportion]

https://doi.org/10.3390/ani16213400

	Periodontal disease	300	Hepatic lipidosis	315
	Hypertrophic cardiomyopathy	270	Periodontal disease	300
2[2]	**Kitten**	**Score**	**Young adult**	**Score**
	Stomatitis	60	Chronic kidney disease	90
	Feline infectious peritonitis	56	Urolithiasis	80
	Feline idiopathic cystitis	56	Pancreatitis	72
	Feline panleukopenia virus infection	50	Systemic hypertension	64
	Gastroenteritis	45	Feline idiopathic cystitis	63
	Ocular disease	40	Hypertrophic cardiomyopathy	63
	Feline leukemia virus infection	35	Hepatic lipidosis	63
	Feline calicivirus infection	32	Stomatitis	60
	Dermatitis	27	Periodontal disease	60
	Feline herpesvirus infection	18	Diabetes mellitus	50
	**Mature adult**	**Score**	**Senior**	**Score**
	Chronic kidney disease	90	Chronic kidney disease	90
	Pancreatitis	90	Pancreatitis	81
	Diabetes mellitus	80	Hyperthyroidism	80
	Systemic hypertension	72	Diabetes mellitus	80
	Urolithiasis	70	Systemic hypertension	80
	Heart failure	70	Hypertrophic cardiomyopathy	63
	Hypertrophic cardiomyopathy	63	Periodontal disease	60
	Hepatic lipidosis	63	Stomatitis	54
	Stomatitis	60	Urolithiasis	50
	Periodontal disease	60	Heart failure	50

[2] Decile [(disease importance × treatment/management efficacy)] × Decile [diagnostic proportion]

https://doi.org/10.3390/ani16213400

Supplementary Table S2. Complete Candidate Diagnostic Proportions and Priority Scores.

These tables report all 32 candidate categories using 39,016 diagnosis entries. Counts describe diagnosis entries, not unique cats. S2A contains the expert-derived category scores used in the model; S2B–S2E report stage-specific diagnosis counts, diagnostic proportions, confidence intervals, and priority scores.

V1 and V2 denote the two priority scores. Eligibility requires an age-specific-to-overall diagnostic proportion ratio of at least 0.5 and a stage-specific diagnostic proportion of at least 0.3%. Values are rounded for display only; eligibility uses unrounded values. N denotes an ineligible candidate whose calculated scores are retained for transparency.

Proportion and confidence limits are percentages of diagnosis entries. Ratio is the age-specific-to-overall diagnostic proportion ratio, not relative risk. Intervals are pointwise 95% hospital-cluster percentile bootstrap intervals from 10,000 resamples of 27 hospitals. They preserve within-hospital dependence but do not correct cross-hospital duplicate cats, selection or coding bias. NE indicates that an informative bootstrap interval cannot be estimated because no diagnoses were observed in any hospital in that stage. These are not cat-prevalence intervals. Scores and eligibility use the point estimates; confidence limits are not scoring inputs.

S2A Expert-derived category scores used in the model

Candidate disease	Importance score	Efficacy score	Valid n per source item
Feline leukemia virus infection	9.17	6.04	16
Feline coronavirus infection	7.22	6.67	12
Feline panleukopenia virus infection	8.89	7.22	18
Feline calicivirus infection	7.96	6.85	18
Feline herpesvirus infection	6.84	7.02	19
Hyperthyroidism	8.71	8.60	31
Megacolon	7.43	7.12	22
Cholelithiasis	6.67	7.29	16
Urolithiasis	9.24	8.19	35
Feline infectious peritonitis	9.73	7.07	25
Osteoarthritis	7.54	6.84	19
Stomatitis	7.81	8.29	35
Polycystic kidney disease	7.82	5.52	32
Diabetes mellitus	9.57	8.38	31
Chronic kidney disease	9.65	7.72	38
Feline idiopathic cystitis	8.76	8.00	35
Cataract	6.00	5.33	10
Infectious anemia	9.21	7.62	21

https://doi.org/10.3390/ani16213400

Candidate disease	Importance score	Efficacy score	Valid n per source item
Hydronephrosis	9.61	7.57	26
Hypertrophic cardiomyopathy	10.00	6.87	32
Heart failure	9.60	7.88	33
Ocular disease	6.35	8.61	4–17
Gastroenteritis	7.76	7.72	17–29
Cognitive dysfunction syndrome	6.67	5.83	4
Systemic hypertension	8.77	8.42	19
Neoplasia	9.30	6.95	11–26
Hepatic lipidosis	9.43	7.90	35
Asthma	8.27	7.60	25
Pancreatitis	9.08	8.16	29
Periodontal disease	7.35	8.96	20–33
Dermatitis	6.04	8.61	13–15
Hairball	5.94	8.26	23

The 10-point model scores were calculated from rounded High/Medium/Low response percentages. For merged categories, each constituent questionnaire item contributed equally. The valid n range refers to individual questionnaire items, not the number of distinct veterinarians contributing to a merged category. Item-level means and sample SDs on the original 15/10/5 scale are reported in Table S3.

S2B Kitten diagnostic denominator 11,965

Candidate disease	Count	Proportion (%) (95% CI)	Ratio	V1	V2	Eligible
Asthma	16	0.134 (0.070–0.191)	0.51	75	15	N
Cataract	36	0.301 (0.048–0.541)	1.16	5	5	Y
Cholelithiasis	7	0.059 (0.000–0.119)	0.23	20	4	N
Chronic kidney disease	37	0.309 (0.181–0.391)	0.10	300	45	N
Cognitive dysfunction syndrome	4	0.033 (0.008–0.072)	0.47	4	2	N

https://doi.org/10.3390/ani16213400

Candidate disease	Count	Proportion (%) (95% CI)	Ratio	V1	V2	Eligible
Dermatitis	217	1.814 (1.324–2.570)	1.77	90	27	Y
Diabetes mellitus	1	0.008 (0.000–0.025)	0.01	81	10	N
Feline calicivirus infection	64	0.535 (0.304–0.836)	2.07	120	32	Y
Feline coronavirus infection	46	0.384 (0.223–0.681)	1.63	36	12	Y
Feline herpesvirus infection	191	1.596 (0.910–2.636)	1.83	108	18	Y
Feline idiopathic cystitis	71	0.593 (0.466–0.689)	0.53	336	56	Y
Feline infectious peritonitis	150	1.254 (0.467–2.219)	2.43	320	56	Y
Feline leukemia virus infection	60	0.501 (0.193–0.888)	2.10	98	35	Y
Feline panleukopenia virus infection	245	2.048 (1.259–2.852)	2.71	350	50	Y
Gastroenteritis	211	1.763 (0.953–2.664)	1.24	216	45	Y
Hairball	45	0.376 (0.280–0.451)	0.55	48	18	Y
Heart failure	5	0.042 (0.000–0.085)	0.12	126	20	N
Hepatic lipidosis	4	0.033 (0.009–0.060)	0.06	126	18	N
Hydronephrosis	16	0.134 (0.017–0.229)	0.34	150	24	N
Hyperthyroidism	1	0.008 (0.000–0.035)	0.03	60	10	N
Hypertrophic cardiomyopathy	48	0.401 (0.194–0.638)	0.22	210	49	N
Infectious anemia	10	0.084 (0.031–0.115)	0.99	144	24	N

https://doi.org/10.3390/ani16213400

Candidate disease	Count	Proportion (%) (95% CI)	Ratio	V1	V2	Eligible
Megacolon	25	0.209 (0.080–0.331)	0.66	80	20	N
Neoplasia	17	0.142 (0.049–0.300)	0.23	96	24	N
Ocular disease	923	7.714 (5.998–10.092)	1.63	200	40	Y
Osteoarthritis	19	0.159 (0.096–0.246)	0.28	32	12	N
Pancreatitis	40	0.334 (0.167–0.471)	0.25	336	54	N
Periodontal disease	347	2.900 (2.016–3.915)	0.44	300	60	N
Polycystic kidney disease	9	0.075 (0.034–0.139)	0.22	15	3	N
Stomatitis	285	2.382 (1.993–3.071)	0.76	450	60	Y
Systemic hypertension	60	0.501 (0.041–1.146)	0.35	378	56	N
Urolithiasis	21	0.176 (0.042–0.395)	0.25	320	50	N

Proportion and confidence limits are percentages of diagnosis entries. Ratio is the age-specific-to-overall diagnostic proportion ratio, not relative risk. Intervals are pointwise 95% hospital-cluster percentile bootstrap intervals from 10,000 resamples of 27 hospitals. They preserve within-hospital dependence but do not correct cross-hospital duplicate cats, selection or coding bias. NE indicates that an informative bootstrap interval cannot be estimated because no diagnoses were observed in any hospital in that stage. These are not cat-prevalence intervals. Scores and eligibility use the point estimates; confidence limits are not scoring inputs.

S2C Young Adult diagnostic denominator 16,022

Candidate disease	Count	Proportion (%) (95% CI)	Ratio	V1	V2	Eligible
Asthma	60	0.374 (0.273–0.478)	1.42	125	25	Y
Cataract	32	0.200 (0.024–0.395)	0.77	3	3	N
Cholelithiasis	32	0.200 (0.096–0.277)	0.79	30	6	N

https://doi.org/10.3390/ani16213400

Candidate disease	Count	Proportion (%) (95% CI)	Ratio	V1	V2	Eligible
Chronic kidney disease	423	2.640 (1.855–3.100)	0.83	600	90	Y
Cognitive dysfunction syndrome	2	0.012 (0.000–0.029)	0.17	2	1	N
Dermatitis	132	0.824 (0.529–1.353)	0.80	70	21	Y
Diabetes mellitus	60	0.374 (0.185–0.618)	0.67	405	50	Y
Feline calicivirus infection	28	0.175 (0.103–0.318)	0.68	30	8	N
Feline coronavirus infection	38	0.237 (0.091–0.427)	1.01	18	6	N
Feline herpesvirus infection	91	0.568 (0.228–1.133)	0.65	72	12	Y
Feline idiopathic cystitis	308	1.922 (1.419–2.199)	1.71	378	63	Y
Feline infectious peritonitis	38	0.237 (0.133–0.364)	0.46	120	21	N
Feline leukemia virus infection	19	0.119 (0.043–0.200)	0.50	28	10	N
Feline panleukopenia virus infection	45	0.281 (0.158–0.428)	0.37	140	20	N
Gastroenteritis	220	1.373 (0.915–1.866)	0.97	216	45	Y
Hairball	141	0.880 (0.731–1.125)	1.28	56	21	Y
Heart failure	50	0.312 (0.163–0.438)	0.86	252	40	Y
Hepatic lipidosis	130	0.811 (0.396–1.117)	1.54	441	63	Y
Hydronephrosis	94	0.587 (0.112–0.971)	1.48	300	48	Y
Hyperthyroidism	4	0.025 (0.000–0.065)	0.08	60	10	N

Candidate disease	Count	Proportion (%) (95% CI)	Ratio	V1	V2	Eligible
Hypertrophic cardiomyopathy	360	2.247 (0.858–3.024)	1.26	270	63	Y
Infectious anemia	11	0.069 (0.000–0.126)	0.81	48	8	N
Megacolon	55	0.343 (0.105–0.636)	1.08	80	20	Y
Neoplasia	39	0.243 (0.127–0.384)	0.39	96	24	N
Ocular disease	634	3.957 (2.886–5.248)	0.84	200	40	Y
Osteoarthritis	97	0.605 (0.332–1.011)	1.08	48	18	Y
Pancreatitis	151	0.942 (0.607–1.117)	0.72	448	72	Y
Periodontal disease	1552	9.687 (6.031–16.516)	1.46	300	60	Y
Polycystic kidney disease	63	0.393 (0.161–0.625)	1.17	25	5	Y
Stomatitis	611	3.814 (2.540–6.074)	1.21	450	60	Y
Systemic hypertension	188	1.173 (0.209–2.276)	0.81	432	64	Y
Urolithiasis	170	1.061 (0.556–1.744)	1.54	512	80	Y

Proportion and confidence limits are percentages of diagnosis entries. Ratio is the age-specific-to-overall diagnostic proportion ratio, not relative risk. Intervals are pointwise 95% hospital-cluster percentile bootstrap intervals from 10,000 resamples of 27 hospitals. They preserve within-hospital dependence but do not correct cross-hospital duplicate cats, selection or coding bias. NE indicates that an informative bootstrap interval cannot be estimated because no diagnoses were observed in any hospital in that stage. These are not cat-prevalence intervals. Scores and eligibility use the point estimates; confidence limits are not scoring inputs.

S2D Mature Adult diagnostic denominator 7,098

Candidate disease	Count	Proportion (%) (95% CI)	Ratio	V1	V2	Eligible
Asthma	21	0.296 (0.123–0.413)	1.12	75	15	N

Candidate disease	Count	Proportion (%) (95% CI)	Ratio	V1	V2	Eligible
Cataract	13	0.183 (0.036–0.380)	0.71	3	3	N
Cholelithiasis	34	0.479 (0.171–0.900)	1.89	50	10	Y
Chronic kidney disease	422	5.945 (4.905–6.946)	1.87	600	90	Y
Cognitive dysfunction syndrome	2	0.028 (0.000–0.063)	0.39	2	1	N
Dermatitis	41	0.578 (0.422–0.825)	0.56	50	15	Y
Diabetes mellitus	90	1.268 (0.687–2.015)	2.26	648	80	Y
Feline calicivirus infection	9	0.127 (0.026–0.273)	0.49	30	8	N
Feline coronavirus infection	5	0.070 (0.020–0.186)	0.30	6	2	N
Feline herpesvirus infection	24	0.338 (0.128–0.722)	0.39	48	8	N
Feline idiopathic cystitis	50	0.704 (0.376–0.846)	0.63	252	42	Y
Feline infectious peritonitis	6	0.085 (0.000–0.190)	0.16	80	14	N
Feline leukemia virus infection	10	0.141 (0.034–0.317)	0.59	42	15	N
Feline panleukopenia virus infection	3	0.042 (0.000–0.089)	0.06	35	5	N
Gastroenteritis	76	1.071 (0.577–1.406)	0.76	192	40	Y
Hairball	53	0.747 (0.459–1.179)	1.09	48	18	Y
Heart failure	69	0.972 (0.603–1.429)	2.69	441	70	Y
Hepatic lipidosis	58	0.817 (0.303–1.537)	1.56	441	63	Y

https://doi.org/10.3390/ani16213400

Candidate disease	Count	Proportion (%) (95% CI)	Ratio	V1	V2	Eligible
Hydronephrosis	34	0.479 (0.112–0.883)	1.21	250	40	Y
Hyperthyroidism	26	0.366 (0.122–0.575)	1.23	240	40	Y
Hypertrophic cardiomyopathy	201	2.832 (1.295–4.497)	1.59	270	63	Y
Infectious anemia	9	0.127 (0.000–0.259)	1.50	96	16	N
Megacolon	33	0.465 (0.055–1.065)	1.46	64	16	Y
Neoplasia	111	1.564 (0.738–3.013)	2.52	192	48	Y
Ocular disease	161	2.268 (1.491–3.251)	0.48	180	36	N
Osteoarthritis	55	0.775 (0.538–1.216)	1.39	48	18	Y
Pancreatitis	210	2.959 (1.785–3.880)	2.25	560	90	Y
Periodontal disease	494	6.960 (4.493–11.958)	1.05	300	60	Y
Polycystic kidney disease	47	0.662 (0.345–0.987)	1.97	25	5	Y
Stomatitis	207	2.916 (2.017–4.618)	0.93	450	60	Y
Systemic hypertension	170	2.395 (0.542–4.275)	1.65	486	72	Y
Urolithiasis	60	0.845 (0.460–1.153)	1.23	448	70	Y

Proportion and confidence limits are percentages of diagnosis entries. Ratio is the age-specific-to-overall diagnostic proportion ratio, not relative risk. Intervals are pointwise 95% hospital-cluster percentile bootstrap intervals from 10,000 resamples of 27 hospitals. They preserve within-hospital dependence but do not correct cross-hospital duplicate cats, selection or coding bias. NE indicates that an informative bootstrap interval cannot be estimated because no diagnoses were observed in any hospital in that stage. These are not cat-prevalence intervals. Scores and eligibility use the point estimates; confidence limits are not scoring inputs.

S2E Senior diagnostic denominator 3,931

Candidate disease	Count	Proportion (%) (95% CI)	Ratio	V1	V2	Eligible
Asthma	6	0.153 (0.050–0.362)	0.58	50	10	N
Cataract	20	0.509 (0.074–0.811)	1.97	6	6	Y
Cholelithiasis	26	0.661 (0.261–0.868)	2.61	60	12	Y
Chronic kidney disease	359	9.133 (7.541–10.740)	2.87	600	90	Y
Cognitive dysfunction syndrome	20	0.509 (0.000–2.283)	7.09	12	6	Y
Dermatitis	10	0.254 (0.091–0.396)	0.25	30	9	N
Diabetes mellitus	68	1.730 (0.632–2.729)	3.08	648	80	Y
Feline calicivirus infection	0	0.000 (NE)	0.00	15	4	N
Feline coronavirus infection	3	0.076 (0.000–0.206)	0.32	12	4	N
Feline herpesvirus infection	34	0.865 (0.055–2.642)	0.99	84	14	Y
Feline idiopathic cystitis	10	0.254 (0.126–0.482)	0.23	126	21	N
Feline infectious peritonitis	7	0.178 (0.027–0.471)	0.35	120	21	N
Feline leukemia virus infection	4	0.102 (0.000–0.202)	0.43	28	10	N
Feline panleukopenia virus infection	2	0.051 (0.000–0.099)	0.07	35	5	N
Gastroenteritis	46	1.170 (0.461–2.650)	0.83	168	35	Y
Hairball	29	0.738 (0.428–1.599)	1.07	48	18	Y
Heart failure	17	0.432 (0.202–0.626)	1.20	315	50	Y
Hepatic lipidosis	13	0.331 (0.077–0.494)	0.63	315	45	Y
Hydronephrosis	11	0.280 (0.144–0.469)	0.70	200	32	N
Hyperthyroidism	85	2.162 (1.081–3.334)	7.27	480	80	Y
Hypertrophic cardiomyopathy	88	2.239 (1.204–2.676)	1.25	270	63	Y
Infectious anemia	3	0.076 (0.000–0.149)	0.90	96	16	N
Megacolon	11	0.280 (0.057–0.734)	0.88	64	16	N
Neoplasia	75	1.908 (1.394–2.747)	3.08	192	48	Y
Ocular disease	124	3.154 (0.681–5.032)	0.67	200	40	Y
Osteoarthritis	47	1.196 (0.439–2.461)	2.14	56	21	Y
Pancreatitis	113	2.875 (1.531–3.666)	2.18	504	81	Y

https://doi.org/10.3390/ani16213400

Candidate disease	Count	Proportion (%) (95% CI)	Ratio	V1	V2	Eligible
Periodontal disease	195	4.961 (3.421–8.430)	0.75	300	60	Y
Polycystic kidney disease	12	0.305 (0.064–0.494)	0.91	20	4	Y
Stomatitis	123	3.129 (2.077–5.408)	1.00	405	54	Y
Systemic hypertension	147	3.740 (1.030–5.732)	2.58	540	80	Y
Urolithiasis	18	0.458 (0.234–0.869)	0.66	320	50	Y

Proportion and confidence limits are percentages of diagnosis entries. Ratio is the age-specific-to-overall diagnostic proportion ratio, not relative risk. Intervals are pointwise 95% hospital-cluster percentile bootstrap intervals from 10,000 resamples of 27 hospitals. They preserve within-hospital dependence but do not correct cross-hospital duplicate cats, selection or coding bias. NE indicates that an informative bootstrap interval cannot be estimated because no diagnoses were observed in any hospital in that stage. These are not cat-prevalence intervals. Scores and eligibility use the point estimates; confidence limits are not scoring inputs.

Supplementary Table S3. Individual Expert Ratings and Descriptive Statistics.

This table covers only the two criteria used in the priority model—disease importance and treatment/management efficacy—and the 46 source questionnaire items contributing to its 32 candidate disease categories. Thirty-nine veterinarians participated.

Respondents are anonymized as V01–V39, and the same identifier denotes the same respondent in every panel. High, Medium, and Low responses are scored 15, 10, and 5, respectively. A dash denotes no recorded score; it is not zero. No missing scores were imputed. Each criterion has 1,007 valid ratings across the 46 items.

S3A and S3B summarize the original individual ratings using the valid item-specific n, High/Medium/Low counts, mean, and sample standard deviation (SD, denominator n−1). S3D and S3E show all individual ratings in three respondent panels per criterion. The exact item means may differ slightly from the model scores after rescaling because the model used rounded response percentages. The category-level model scores are reported separately in S2A.

S3A Disease importance

Questionnaire item	Valid n	High	Medium	Low	Mean	SD
Keratitis	12	4	5	3	10.42	3.96
Hepatic neoplasia	20	15	5	0	13.75	2.22
Hyperthyroidism	31	19	12	0	13.06	2.48
Megacolon	22	8	11	3	11.14	3.43
Keratoconjunctivitis sicca	6	1	3	2	9.17	3.76
Conjunctivitis	17	2	8	7	8.53	3.43
Feline infectious peritonitis (FIP)	25	23	2	0	14.60	1.38
Feline calicivirus (FCV) infection	18	8	9	1	11.94	3.04
Feline idiopathic cystitis	35	23	11	1	13.14	2.73
Feline panleukopenia (FPV infection)	18	12	6	0	13.33	2.43
Feline herpesvirus infection (viral rhinotracheitis)	19	6	8	5	10.26	3.90
Feline hairballs (trichobezoars)	23	2	14	7	8.91	3.00
Feline leukemia virus (FeLV) infection	16	12	4	0	13.75	2.24
Feline coronavirus (FCoV) infection	12	4	6	2	10.83	3.59
Osteoarthritis	19	7	10	2	11.32	3.27
Oral squamous cell carcinoma	11	10	1	0	14.55	1.51
Stomatitis	35	13	21	1	11.71	2.70
Polycystic kidney disease	32	14	15	3	11.72	3.27
Cholelithiasis	16	2	12	2	10.00	2.58

Questionnaire item	Valid n	High	Medium	Low	Mean	SD
Diabetes mellitus	31	27	4	0	14.35	1.70
Lymphoma	26	25	1	0	14.81	0.98
Chronic kidney disease	38	34	4	0	14.47	1.56
Cataract	10	1	6	3	9.00	3.16
Nasal/paranasal sinus carcinoma	18	15	3	0	14.17	1.92
Hypertrophic cardiomyopathy	32	32	0	0	15.00	0.00
Splenic neoplasia	19	12	5	2	12.63	3.48
Anemia (all causes)	21	17	3	1	13.81	2.69
Alimentary lymphoma	23	21	2	0	14.57	1.44
Hydronephrosis	26	24	1	1	14.42	2.16
Heart failure	33	29	4	0	14.39	1.66
Allergic dermatitis	13	1	7	5	8.46	3.15
Inflammatory bowel disease	29	14	15	0	12.41	2.54
Urolithiasis	35	27	8	0	13.86	2.13
Gastroenteritis	17	5	10	2	10.88	3.18
Epiphora	4	1	2	1	10.00	4.08
Mammary neoplasia	24	18	5	1	13.54	2.75
Cognitive dysfunction syndrome	4	2	0	2	10.00	5.77
Systemic hypertension	19	12	7	0	13.16	2.48
Hepatic lipidosis	35	29	6	0	14.14	1.91
Asthma	25	12	13	0	12.40	2.55
Pancreatitis	29	21	8	0	13.62	2.27
Dental calculus	20	5	9	6	9.75	3.80
Tooth resorptive lesions	30	12	15	3	11.50	3.26
Periodontal disease	33	15	15	3	11.82	3.26
Dermatophytosis	15	3	8	4	9.67	3.52
Subcutaneous fibrosarcoma	11	8	3	0	13.64	2.34

Scores are on the original 15/10/5 scale. Statistics use observed ratings only. Item labels and the combined model categories are distinguished in S3C. The category-level analysis scores in S2A were calculated from rounded response percentages.

https://doi.org/10.3390/ani16213400

S3B Treatment and management efficacy

Questionnaire item	Valid n	High	Medium	Low	Mean	SD
Keratitis	12	10	1	1	13.75	3.11
Hepatic neoplasia	20	7	9	4	10.75	3.73
Hyperthyroidism	31	18	13	0	12.90	2.51
Megacolon	22	5	15	2	10.68	2.80
Keratoconjunctivitis sicca	6	4	2	0	13.33	2.58
Conjunctivitis	17	9	6	2	12.06	3.56
Feline infectious peritonitis (FIP)	25	11	6	8	10.60	4.41
Feline calicivirus (FCV) infection	18	4	11	3	10.28	3.20
Feline idiopathic cystitis	35	16	17	2	12.00	3.02
Feline panleukopenia (FPV infection)	18	7	7	4	10.83	3.93
Feline herpesvirus infection (viral rhinotracheitis)	19	5	11	3	10.53	3.29
Feline hairballs (trichobezoars)	23	14	6	3	12.39	3.65
Feline leukemia virus (FeLV) infection	16	4	5	7	9.06	4.17
Feline coronavirus (FCoV) infection	12	3	6	3	10.00	3.69
Osteoarthritis	19	2	16	1	10.26	2.02
Oral squamous cell carcinoma	11	1	8	2	9.55	2.70
Stomatitis	35	19	14	2	12.43	3.06
Polycystic kidney disease	32	4	13	15	8.28	3.50
Cholelithiasis	16	4	11	1	10.94	2.72
Diabetes mellitus	31	17	13	1	12.58	2.85
Lymphoma	26	3	16	7	9.23	3.06
Chronic kidney disease	38	13	24	1	11.58	2.63
Cataract	10	1	4	5	8.00	3.50
Nasal/paranasal sinus carcinoma	18	3	7	8	8.61	3.76
Hypertrophic cardiomyopathy	32	8	18	6	10.31	3.35
Splenic neoplasia	19	12	5	2	12.63	3.48
Anemia (all causes)	21	7	13	1	11.43	2.80
Alimentary lymphoma	23	4	13	6	9.57	3.34

Questionnaire item	Valid n	High	Medium	Low	Mean	SD
Hydronephrosis	26	8	17	1	11.35	2.67
Heart failure	33	13	19	1	11.82	2.74
Allergic dermatitis	13	4	8	1	11.15	3.00
Inflammatory bowel disease	29	8	16	5	10.52	3.37
Urolithiasis	35	18	15	2	12.29	3.05
Gastroenteritis	17	10	6	1	12.65	3.12
Epiphora	4	2	2	0	12.50	2.89
Mammary neoplasia	24	13	6	5	11.67	4.08
Cognitive dysfunction syndrome	4	0	3	1	8.75	2.50
Systemic hypertension	19	10	9	0	12.63	2.56
Hepatic lipidosis	35	16	16	3	11.86	3.23
Asthma	25	8	16	1	11.40	2.71
Pancreatitis	29	14	14	1	12.24	2.86
Dental calculus	20	17	2	1	14.00	2.62
Tooth resorptive lesions	30	18	12	0	13.00	2.49
Periodontal disease	33	22	11	0	13.33	2.39
Dermatophytosis	15	14	1	0	14.67	1.29
Subcutaneous fibrosarcoma	11	5	4	2	11.36	3.93

Scores are on the original 15/10/5 scale. Statistics use observed ratings only. Item labels and the combined model categories are distinguished in S3C. The category-level analysis scores in S2A were calculated from rounded response percentages.

S3C Questionnaire items combined into model categories

Model category	Questionnaire items
Ocular disease	Keratitis; Keratoconjunctivitis sicca; Conjunctivitis; Epiphora
Gastroenteritis	Inflammatory bowel disease; Gastroenteritis
Neoplasia	Hepatic neoplasia; Oral squamous cell carcinoma; Lymphoma; Nasal/paranasal sinus carcinoma; Splenic neoplasia; Alimentary lymphoma; Mammary neoplasia; Subcutaneous fibrosarcoma
Periodontal disease	Dental calculus; Tooth resorptive lesions; Periodontal disease
Dermatitis	Allergic dermatitis; Dermatophytosis

For merged categories, the constituent item scores were averaged with equal weight; individual ratings were not pooled across items.

S3D(a) Disease importance Respondents V01 to V13

Questionnaire item	V01	V02	V03	V04	V05	V06	V07	V08	V09	V10	V11	V12	V13
Keratitis	—	10	—	—	—	—	10	10	—	—	5	5	—
Hepatic neoplasia	15	15	—	—	—	15	—	10	10	10	10	—	—
Hyperthyroidism	—	10	15	15	15	15	15	10	—	10	—	10	15
Megacolon	—	10	15	—	5	10	10	—	10	5	—	—	—
Keratoconjunctivitis sicca	—	—	—	—	—	—	—	—	—	—	5	15	—
Conjunctivitis	—	5	—	—	—	—	10	10	—	—	5	5	10
Feline infectious peritonitis (FIP)	15	—	15	15	15	15	15	15	—	15	—	—	15
Feline calicivirus (FCV) infection	15	—	—	15	10	—	10	—	—	15	—	—	10
Feline idiopathic cystitis	15	—	15	15	10	15	10	15	15	10	10	15	15
Feline panleukopenia (FPV infection)	10	—	15	15	10	15	—	—	—	15	—	—	15
Feline herpesvirus infection (viral rhinotracheitis)	15	5	—	15	10	—	10	—	—	15	—	—	5
Feline hairballs (trichobezoars)	10	—	—	10	—	—	10	10	10	—	5	—	10
Feline leukemia virus (FeLV) infection	15	—	15	10	10	15	15	—	—	15	—	—	—
Feline coronavirus (FCoV) infection	15	—	5	—	10	—	10	—	—	15	—	—	—
Osteoarthritis	15	10	15	—	15	—	—	—	10	—	—	10	—
Oral squamous cell carcinoma	—	—	—	15	15	—	—	—	10	15	—	—	—
Stomatitis	—	—	15	10	15	—	10	10	15	15	10	10	15
Polycystic kidney disease	10	5	10	10	10	15	10	—	10	—	—	10	—
Cholelithiasis	10	—	—	—	—	—	—	10	10	—	10	10	—
Diabetes mellitus	—	15	15	15	15	15	15	15	—	10	10	15	—
Lymphoma	15	—	15	15	15	—	—	15	10	15	15	—	15
Chronic kidney disease	15	15	15	15	15	15	15	15	10	10	10	15	15
Cataract	—	5	—	—	—	—	—	10	—	—	—	10	—
Nasal/paranasal sinus carcinoma	—	—	—	—	15	—	—	—	10	—	—	—	10

https://doi.org/10.3390/ani16213400

Questionnaire item	V01	V02	V03	V04	V05	V06	V07	V08	V09	V10	V11	V12	V13
Hypertrophic cardiomyopathy	15	15	15	15	15	15	15	15	15	—	—	15	15
Splenic neoplasia	15	15	—	—	—	15	—	—	10	5	10	10	—
Anemia (all causes)	—	—	—	15	15	15	—	15	—	10	10	—	10
Alimentary lymphoma	—	—	15	15	15	15	—	—	15	15	15	—	10
Hydronephrosis	15	15	15	15	15	15	—	—	15	—	—	15	—
Heart failure	15	15	15	15	—	15	—	15	15	—	10	15	—
Allergic dermatitis	—	—	—	10	—	10	10	5	—	—	—	10	5
Inflammatory bowel disease	15	—	15	—	10	10	10	10	15	10	10	—	10
Urolithiasis	15	15	15	15	—	15	10	15	15	15	10	15	15
Gastroenteritis	10	—	10	—	—	—	10	10	—	—	5	—	15
Epiphora	—	5	—	—	—	—	—	—	—	—	10	—	—
Mammary neoplasia	—	15	10	—	15	—	—	—	15	15	10	10	15
Cognitive dysfunction syndrome	—	—	—	—	—	—	15	—	—	—	—	—	—
Systemic hypertension	—	15	—	—	15	15	—	15	10	—	—	—	—
Hepatic lipidosis	15	15	15	15	10	15	15	15	10	—	10	—	15
Asthma	—	10	15	—	15	10	10	15	—	—	—	15	10
Pancreatitis	15	15	10	—	15	15	—	15	—	15	10	15	—
Dental calculus	—	5	—	10	—	—	—	—	15	—	5	10	—
Tooth resorptive lesions	10	—	15	15	10	—	—	—	15	15	5	15	10
Periodontal disease	10	15	10	10	15	—	10	10	—	15	10	15	15
Dermatophytosis	—	—	—	—	—	10	10	—	—	—	—	10	5
Subcutaneous fibrosarcoma	—	15	—	—	—	15	—	—	15	10	—	—	—

V01–V39 are anonymous respondent identifiers shared across all panels. High = 15, Medium = 10, Low = 5; — = no recorded score. Missing ratings were not imputed.

S3D(b) Disease importance Respondents V14 to V26

Questionnaire item	V14	V15	V16	V17	V18	V19	V20	V21	V22	V23	V24	V25	V26
Keratitis	—	15	—	—	—	—	15	—	—	—	—	5	—
Hepatic neoplasia	—	15	—	15	—	15	—	15	—	—	—	—	—
Hyperthyroidism	10	—	15	10	15	—	15	15	15	15	15	10	15
Megacolon	15	—	15	15	—	15	10	—	10	—	10	15	5
Keratoconjunctivitis sicca	—	10	—	—	—	—	—	—	—	—	—	—	—
Conjunctivitis	—	10	—	—	—	—	10	—	—	—	15	5	10
Feline infectious peritonitis (FIP)	15	—	15	—	—	—	15	15	15	15	15	—	—
Feline calicivirus (FCV) infection	10	15	10	—	—	—	15	15	—	10	15	10	—
Feline idiopathic cystitis	—	—	15	15	15	15	15	5	10	15	15	15	10
Feline panleukopenia (FPV infection)	—	10	—	—	—	—	—	15	15	15	15	—	—
Feline herpesvirus infection (viral rhinotracheitis)	10	—	10	—	—	—	—	—	10	5	15	5	—
Feline hairballs (trichobezoars)	—	10	10	5	—	15	10	—	—	—	10	5	5
Feline leukemia virus (FeLV) infection	—	15	—	—	—	—	15	15	15	—	15	—	—
Feline coronavirus (FCoV) infection	—	—	—	—	5	—	10	—	—	10	15	—	—
Osteoarthritis	5	15	—	10	15	10	—	—	—	10	—	—	—
Oral squamous cell carcinoma	—	—	—	15	—	—	—	15	—	15	—	—	15
Stomatitis	15	10	15	10	15	15	10	5	—	10	10	10	10
Polycystic kidney disease	15	15	10	15	15	15	—	10	15	10	15	15	5
Cholelithiasis	10	10	—	15	5	5	—	—	10	—	—	—	10
Diabetes mellitus	15	—	15	—	15	—	15	15	15	15	—	15	15
Lymphoma	15	—	—	15	—	15	15	15	15	15	—	—	15
Chronic kidney disease	15	15	15	15	15	15	15	15	15	15	15	15	15
Cataract	5	10	—	—	—	—	—	—	10	—	—	—	—
Nasal/paranasal sinus carcinoma	—	—	15	15	15	15	—	15	—	15	—	15	15

Questionnaire item	V14	V15	V16	V17	V18	V19	V20	V21	V22	V23	V24	V25	V26
Hypertrophic cardiomyopathy	15	—	15	—	15	15	15	—	15	15	15	15	15
Splenic neoplasia	—	—	—	15	10	10	—	—	—	—	—	—	15
Anemia (all causes)	15	—	—	15	15	—	—	15	15	15	15	—	—
Alimentary lymphoma	—	—	15	15	—	10	15	15	—	15	—	15	15
Hydronephrosis	15	15	—	15	15	15	15	15	—	—	15	15	15
Heart failure	15	15	15	—	15	15	10	15	15	15	15	15	15
Allergic dermatitis	—	—	15	—	—	—	—	—	—	—	—	—	—
Inflammatory bowel disease	—	—	15	15	15	10	10	—	15	10	—	15	—
Urolithiasis	10	15	15	15	15	15	15	15	10	—	15	—	15
Gastroenteritis	10	15	—	—	15	—	—	5	—	—	10	10	10
Epiphora	—	—	—	—	—	—	—	—	—	—	15	—	—
Mammary neoplasia	15	15	—	15	—	15	—	15	15	15	—	—	—
Cognitive dysfunction syndrome	—	—	—	—	—	—	—	—	—	—	—	—	—
Systemic hypertension	15	15	—	—	15	10	10	—	15	—	—	15	—
Hepatic lipidosis	15	10	15	15	15	10	15	15	15	15	15	15	15
Asthma	15	—	15	—	15	—	—	15	—	10	—	10	10
Pancreatitis	—	15	10	—	—	—	15	—	15	15	15	15	15
Dental calculus	10	10	—	5	15	15	—	—	—	—	—	10	10
Tooth resorptive lesions	10	15	15	10	15	5	10	15	10	5	—	10	—
Periodontal disease	5	10	15	10	15	15	15	5	10	—	—	10	10
Dermatophytosis	—	10	15	—	10	—	—	—	—	—	10	—	—
Subcutaneous fibrosarcoma	—	—	15	15	—	10	—	15	—	—	10	—	—

V01–V39 are anonymous respondent identifiers shared across all panels. High = 15, Medium = 10, Low = 5; — = no recorded score. Missing ratings were not imputed.

https://doi.org/10.3390/ani16213400

S3D(c) Disease importance Respondents V27 to V39

**Questionnaire item**	**V27**	**V28**	**V29**	**V30**	**V31**	**V32**	**V33**	**V34**	**V35**	**V36**	**V37**	**V38**	**V39**
Keratitis	—	—	—	—	10	—	—	—	15	15	—	10	—
Hepatic neoplasia	10	15	—	15	15	15	—	15	15	15	15	—	—
Hyperthyroidism	10	10	15	10	—	10	15	10	15	—	15	—	15
Megacolon	15	10	—	—	—	—	10	10	15	10	—	—	—
Keratoconjunctivitis sicca	—	5	—	—	10	—	—	—	—	—	—	10	—
Conjunctivitis	5	—	—	—	10	5	—	—	15	—	—	10	5
Feline infectious peritonitis (FIP)	10	—	15	15	—	—	15	—	15	15	15	10	15
Feline calicivirus (FCV) infection	—	—	10	—	—	—	—	—	15	—	—	10	5
Feline idiopathic cystitis	15	15	15	10	15	—	15	10	15	10	10	10	15
Feline panleukopenia (FPV infection)	10	—	15	—	—	—	—	—	15	10	15	10	—
Feline herpesvirus infection (viral rhinotracheitis)	—	—	10	5	—	—	—	—	15	10	—	15	10
Feline hairballs (trichobezoars)	—	10	—	—	10	5	—	5	15	5	—	10	10
Feline leukemia virus (FeLV) infection	10	—	—	—	—	—	—	—	15	15	—	10	—
Feline coronavirus (FCoV) infection	—	—	—	—	—	—	—	—	10	15	—	10	—
Osteoarthritis	—	5	15	—	10	10	—	10	15	10	—	—	—
Oral squamous cell carcinoma	—	—	15	—	—	—	—	—	15	—	15	—	—
Stomatitis	10	10	10	10	15	10	10	10	15	15	10	10	15
Polycystic kidney disease	15	15	15	15	10	10	5	—	10	10	15	10	—
Cholelithiasis	—	10	—	10	—	10	—	—	15	—	—	—	—
Diabetes mellitus	15	15	15	15	15	10	15	15	15	—	15	10	15
Lymphoma	15	15	15	15	—	15	15	—	15	—	15	—	15
Chronic kidney disease	15	15	15	15	15	—	15	15	15	10	15	15	15
Cataract	—	5	—	—	10	10	—	—	15	—	—	—	—
Nasal/paranasal sinus carcinoma	—	—	15	15	—	15	15	—	15	10	15	—	—

https://doi.org/10.3390/ani16213400

Questionnaire item	V27	V28	V29	V30	V31	V32	V33	V34	V35	V36	V37	V38	V39
Hypertrophic cardiomyopathy	15	15	15	15	—	15	15	15	15	—	15	15	15
Splenic neoplasia	—	15	—	15	15	15	—	15	15	—	15	—	5
Anemia (all causes)	15	15	15	—	—	—	15	—	15	—	15	—	5
Alimentary lymphoma	15	15	15	15	—	—	15	—	15	—	15	—	—
Hydronephrosis	15	15	15	15	15	—	10	—	—	—	15	—	5
Heart failure	10	15	15	15	15	15	15	15	15	—	15	10	15
Allergic dermatitis	—	—	—	—	10	10	—	5	—	10	5	—	5
Inflammatory bowel disease	10	10	—	10	15	15	15	10	15	10	15	—	15
Urolithiasis	15	15	15	10	15	15	10	10	15	10	—	15	15
Gastroenteritis	—	10	—	—	15	—	—	—	15	—	—	10	—
Epiphora	—	—	—	—	10	—	—	—	—	—	—	—	—
Mammary neoplasia	—	15	—	15	15	10	—	15	15	15	—	5	10
Cognitive dysfunction syndrome	—	—	—	—	—	—	—	5	15	—	—	5	—
Systemic hypertension	—	10	—	10	—	—	10	15	15	—	—	15	10
Hepatic lipidosis	15	15	15	15	—	15	15	15	15	10	15	15	—
Asthma	10	15	15	10	—	15	15	10	10	10	—	—	10
Pancreatitis	10	15	15	10	—	10	10	15	15	15	15	10	15
Dental calculus	—	5	—	—	10	10	5	5	15	10	—	—	15
Tooth resorptive lesions	10	10	15	10	10	—	10	15	15	10	—	—	10
Periodontal disease	15	5	15	—	10	10	10	15	15	10	—	15	15
Dermatophytosis	—	—	—	—	10	10	—	5	15	5	5	15	—
Subcutaneous fibrosarcoma	—	—	—	—	—	—	—	—	15	—	15	—	—

V01–V39 are anonymous respondent identifiers shared across all panels. High = 15, Medium = 10, Low = 5; — = no recorded score. Missing ratings were not imputed.

S3E(a) Treatment and management efficacy Respondents V01 to V13

**Questionnaire item**	**V01**	**V02**	**V03**	**V04**	**V05**	**V06**	**V07**	**V08**	**V09**	**V10**	**V11**	**V12**	**V13**
Keratitis	—	15	—	—	—	—	10	5	—	—	15	15	—
Hepatic neoplasia	15	5	—	—	—	15	—	10	10	10	10	—	—
Hyperthyroidism	—	15	15	10	15	15	10	10	—	10	—	15	10
Megacolon	—	10	10	—	10	10	10	—	10	10	—	—	—
Keratoconjunctivitis sicca	—	—	—	—	—	—	—	—	—	—	15	15	—
Conjunctivitis	—	15	—	—	—	—	10	10	—	—	15	15	10
Feline infectious peritonitis (FIP)	15	—	15	5	15	15	5	5	—	5	—	—	5
Feline calicivirus (FCV) infection	15	—	—	5	15	—	10	—	—	5	—	—	10
Feline idiopathic cystitis	10	—	15	10	10	5	10	10	10	10	10	10	10
Feline panleukopenia (FPV infection)	15	—	5	10	15	15	—	—	—	5	—	—	10
Feline herpesvirus infection (viral rhinotracheitis)	15	15	—	10	10	—	10	—	—	5	—	—	5
Feline hairballs (trichobezoars)	15	—	—	10	—	—	10	10	15	—	15	—	15
Feline leukemia virus (FeLV) infection	15	—	5	10	5	5	5	—	—	5	—	—	—
Feline coronavirus (FCoV) infection	15	—	10	—	10	—	10	—	—	5	—	—	—
Osteoarthritis	10	5	10	—	10	—	—	—	10	—	—	10	—
Oral squamous cell carcinoma	—	—	—	10	15	—	—	—	10	10	—	—	—
Stomatitis	—	—	10	10	15	—	10	15	10	10	15	15	15
Polycystic kidney disease	15	10	5	10	5	10	10	—	10	—	—	5	—
Cholelithiasis	15	—	—	—	—	—	—	10	10	—	10	10	—
Diabetes mellitus	—	15	15	10	15	15	10	10	—	10	10	15	—
Lymphoma	15	—	10	10	15	—	—	10	10	5	5	—	10
Chronic kidney disease	10	15	15	10	15	10	10	10	10	10	10	10	10
Cataract	—	5	—	—	—	—	—	10	—	—	—	5	—
Nasal/paranasal sinus carcinoma	—	—	—	—	15	—	—	—	5	—	—	—	15

https://doi.org/10.3390/ani16213400

Questionnaire item	V01	V02	V03	V04	V05	V06	V07	V08	V09	V10	V11	V12	V13
Hypertrophic cardiomyopathy	5	15	10	10	5	10	10	10	10	—	—	15	5
Splenic neoplasia	15	15	—	—	—	15	—	—	10	15	10	15	—
Anemia (all causes)	—	—	—	10	10	10	—	10	—	10	10	—	10
Alimentary lymphoma	—	—	10	10	15	10	—	—	15	10	5	—	10
Hydronephrosis	10	10	15	10	15	10	—	—	10	—	—	15	—
Heart failure	10	15	15	10	—	10	—	10	10	—	10	15	—
Allergic dermatitis	—	—	—	10	—	5	10	10	—	—	—	10	15
Inflammatory bowel disease	10	—	10	—	10	5	10	10	10	5	10	—	10
Urolithiasis	15	15	10	10	—	5	10	10	10	10	15	15	10
Gastroenteritis	15	—	10	—	—	—	15	10	—	—	15	—	10
Epiphora	—	15	—	—	—	—	—	—	—	—	10	—	—
Mammary neoplasia	—	5	10	—	15	—	—	—	10	15	15	15	10
Cognitive dysfunction syndrome	—	—	—	—	—	—	10	—	—	—	—	—	—
Systemic hypertension	—	15	—	—	15	10	—	10	10	—	—	—	—
Hepatic lipidosis	15	15	15	10	10	15	10	10	10	—	10	—	15
Asthma	—	15	10	—	15	10	10	10	—	—	—	15	10
Pancreatitis	10	10	10	—	15	10	—	10	—	10	15	15	—
Dental calculus	—	15	—	15	—	—	—	—	15	—	15	15	—
Tooth resorptive lesions	15	—	10	10	10	—	—	—	10	10	10	15	15
Periodontal disease	15	15	10	15	15	—	15	10	—	10	15	15	15
Dermatophytosis	—	—	—	—	—	15	15	—	—	—	—	15	15
Subcutaneous fibrosarcoma	—	15	—	—	—	15	—	—	10	5	—	—	—

V01–V39 are anonymous respondent identifiers shared across all panels. High = 15, Medium = 10, Low = 5; — = no recorded score. Missing ratings were not imputed.

S3E(b) Treatment and management efficacy Respondents V14 to V26

Questionnaire item	V14	V15	V16	V17	V18	V19	V20	V21	V22	V23	V24	V25	V26
Keratitis	—	15	—	—	—	—	15	—	—	—	—	15	—
Hepatic neoplasia	—	15	—	15	—	10	—	5	—	—	—	—	—
Hyperthyroidism	15	—	15	10	15	—	10	15	15	10	15	10	15
Megacolon	10	—	15	10	—	5	10	—	10	—	15	15	5
Keratoconjunctivitis sicca	—	15	—	—	—	—	—	—	—	—	—	—	—
Conjunctivitis	—	10	—	—	—	—	15	—	—	—	15	15	5
Feline infectious peritonitis (FIP)	10	—	15	—	—	—	10	5	5	15	15	—	—
Feline calicivirus (FCV) infection	10	10	10	—	—	—	10	10	—	10	15	15	—
Feline idiopathic cystitis	—	—	15	10	10	15	15	15	10	15	15	15	15
Feline panleukopenia (FPV infection)	—	10	—	—	—	—	—	10	10	15	15	—	—
Feline herpesvirus infection (viral rhinotracheitis)	10	—	10	—	—	—	—	—	10	10	15	15	—
Feline hairballs (trichobezoars)	—	15	10	10	—	15	15	—	—	—	15	15	5
Feline leukemia virus (FeLV) infection	—	15	—	—	—	—	10	10	10	—	15	—	—
Feline coronavirus (FCoV) infection	—	—	—	—	5	—	10	—	—	10	15	—	—
Osteoarthritis	10	15	—	10	15	10	—	—	—	10	—	—	—
Oral squamous cell carcinoma	—	—	—	10	—	—	—	10	—	10	—	—	5
Stomatitis	5	15	15	15	15	15	15	15	—	10	15	15	10
Polycystic kidney disease	15	10	10	5	5	5	—	5	10	10	15	5	5
Cholelithiasis	10	15	—	15	10	10	—	—	10	—	—	—	5
Diabetes mellitus	15	—	15	—	15	—	15	10	10	15	—	10	15
Lymphoma	10	—	—	10	—	5	10	10	10	10	—	—	10
Chronic kidney disease	15	10	15	10	15	10	10	10	10	10	15	10	15
Cataract	15	5	—	—	—	—	—	—	10	—	—	—	—
Nasal/paranasal sinus carcinoma	—	—	15	10	5	5	—	5	—	10	—	5	5

https://doi.org/10.3390/ani16213400

Questionnaire item	V14	V15	V16	V17	V18	V19	V20	V21	V22	V23	V24	V25	V26
Hypertrophic cardiomyopathy	15	—	15	—	10	10	5	—	10	10	15	10	5
Splenic neoplasia	—	—	—	15	15	10	—	—	—	—	—	—	5
Anemia (all causes)	10	—	—	10	10	—	—	10	10	15	15	—	—
Alimentary lymphoma	—	—	15	10	—	10	10	10	—	10	—	5	10
Hydronephrosis	10	10	—	15	15	10	10	10	—	—	15	5	10
Heart failure	15	10	15	—	15	15	15	10	10	10	15	10	15
Allergic dermatitis	—	—	15	—	—	—	—	—	—	—	—	—	—
Inflammatory bowel disease	—	—	15	15	15	5	15	—	10	10	—	5	—
Urolithiasis	10	10	15	15	10	15	10	15	15	—	15	—	5
Gastroenteritis	15	15	—	—	15	—	—	15	—	—	15	15	5
Epiphora	—	—	—	—	—	—	—	—	—	—	15	—	—
Mammary neoplasia	5	15	—	15	—	5	—	15	15	10	—	—	—
Cognitive dysfunction syndrome	—	—	—	—	—	—	—	—	—	—	—	—	—
Systemic hypertension	15	15	—	—	15	10	10	—	10	—	—	10	—
Hepatic lipidosis	10	15	15	10	15	10	15	10	10	15	15	5	10
Asthma	10	—	15	—	15	—	—	10	—	15	—	10	10
Pancreatitis	—	15	15	—	—	—	10	—	10	10	15	10	15
Dental calculus	15	15	—	15	15	15	—	—	—	—	—	15	5
Tooth resorptive lesions	15	15	15	15	15	10	10	15	15	15	—	15	—
Periodontal disease	15	15	15	15	15	15	10	15	15	—	—	15	10
Dermatophytosis	—	15	15	—	15	—	—	—	—	—	15	—	—
Subcutaneous fibrosarcoma	—	—	10	15	—	15	—	10	—	—	15	—	—

V01–V39 are anonymous respondent identifiers shared across all panels. High = 15, Medium = 10, Low = 5; — = no recorded score. Missing ratings were not imputed.

S3E(c) Treatment and management efficacy Respondents V27 to V39

Questionnaire item	V27	V28	V29	V30	V31	V32	V33	V34	V35	V36	V37	V38	V39
Keratitis	—	—	—	—	15	—	—	—	15	15	—	15	—
Hepatic neoplasia	10	10	—	15	15	10	—	15	10	5	5	—	—
Hyperthyroidism	10	10	15	10	—	10	15	15	15	—	15	—	15
Megacolon	10	10	—	—	—	—	10	15	15	10	—	—	—
Keratoconjunctivitis sicca	—	10	—	—	15	—	—	—	—	—	—	10	—
Conjunctivitis	10	—	—	—	15	15	—	—	15	—	—	10	5
Feline infectious peritonitis (FIP)	10	—	15	10	—	—	10	—	15	10	15	15	5
Feline calicivirus (FCV) infection	—	—	10	—	—	—	—	—	10	—	—	10	5
Feline idiopathic cystitis	15	15	10	15	15	—	15	15	10	10	15	10	5
Feline panleukopenia (FPV infection)	5	—	15	—	—	—	—	—	15	10	5	10	—
Feline herpesvirus infection (viral rhinotracheitis)	—	—	10	15	—	—	—	—	10	5	—	10	10
Feline hairballs (trichobezoars)	—	15	—	—	15	15	—	15	5	5	—	10	15
Feline leukemia virus (FeLV) infection	5	—	—	—	—	—	—	—	5	15	—	10	—
Feline coronavirus (FCoV) infection	—	—	—	—	—	—	—	—	5	15	—	10	—
Osteoarthritis	—	10	10	—	10	10	—	10	10	10	—	—	—
Oral squamous cell carcinoma	—	—	10	—	—	—	—	—	10	—	5	—	—
Stomatitis	10	15	10	10	10	10	10	15	15	15	15	10	5
Polycystic kidney disease	5	10	10	5	10	5	5	—	5	15	5	10	—
Cholelithiasis	—	15	—	10	—	10	—	—	10	—	—	—	—
Diabetes mellitus	10	15	15	15	15	10	10	15	10	—	15	5	10
Lymphoma	10	15	5	10	—	5	10	—	10	—	5	—	5
Chronic kidney disease	5	10	10	10	10	—	15	15	15	15	10	10	15
Cataract	—	5	—	—	10	5	—	—	10	—	—	—	—
Nasal/paranasal sinus carcinoma	—	—	5	10	—	10	10	—	10	10	5	—	—

Questionnaire item	V27	V28	V29	V30	V31	V32	V33	V34	V35	V36	V37	V38	V39
Hypertrophic cardiomyopathy	5	10	10	10	—	10	10	15	10	—	10	15	15
Splenic neoplasia	—	15	—	10	15	5	—	15	15	—	15	—	10
Anemia (all causes)	10	15	15	—	—	—	15	—	15	—	15	—	5
Alimentary lymphoma	5	10	5	10	—	—	5	—	15	—	5	—	—
Hydronephrosis	10	10	10	15	15	—	10	—	—	—	10	—	10
Heart failure	10	10	10	10	10	10	10	15	15	—	10	15	5
Allergic dermatitis	—	—	—	—	10	10	—	15	—	10	15	—	10
Inflammatory bowel disease	10	10	—	5	15	10	10	15	15	15	10	—	10
Urolithiasis	15	10	15	10	15	15	10	15	15	15	—	15	10
Gastroenteritis	—	10	—	—	15	—	—	—	10	—	—	10	—
Epiphora	—	—	—	—	10	—	—	—	—	—	—	—	—
Mammary neoplasia	—	15	—	10	15	15	—	5	15	15	—	5	10
Cognitive dysfunction syndrome	—	—	—	—	—	—	—	10	10	—	—	5	—
Systemic hypertension	—	10	—	15	—	—	10	15	15	—	—	15	15
Hepatic lipidosis	5	15	10	10	—	15	10	15	10	5	15	15	—
Asthma	10	10	10	10	—	15	10	15	5	10	—	—	10
Pancreatitis	10	15	15	10	—	10	10	15	15	15	15	5	15
Dental calculus	—	15	—	—	15	15	15	10	15	15	—	—	10
Tooth resorptive lesions	10	15	15	10	15	—	10	15	15	10	—	—	15
Periodontal disease	10	10	15	—	10	10	10	15	15	15	—	10	15
Dermatophytosis	—	—	—	—	15	15	—	15	15	15	15	10	—
Subcutaneous fibrosarcoma	—	—	—	—	—	—	—	—	10	—	5	—	—

V01–V39 are anonymous respondent identifiers shared across all panels. High = 15, Medium = 10, Low = 5; — = no recorded score. Missing ratings were not imputed.

Disclaimer/Publisher's Note: The statements, opinions and data contained in all publications are solely those of the individual author(s) and contributor(s) and not of MDPI and/or the editor(s). MDPI and/or the editor(s) disclaim responsibility for any injury to people or property resulting from any ideas, methods, instructions or products referred to in the content.
